# Cerebral Venous Thrombosis in Pediatric Oncology: A Case of Burkitt Lymphoma

**DOI:** 10.7759/cureus.90686

**Published:** 2025-08-21

**Authors:** Khalil Elouadghiri Fouad, Ayad Ghanam, Manal Azizi, Mohammed Leknani, Imane Kamaoui, Imane Skiker, Abdeladim Babakhouya, Maria Rkain

**Affiliations:** 1 Department of Pediatrics, Faculty of Medicine and Pharmacy, Mohamed First University, Oujda, MAR; 2 Department of Pediatrics, Mohammed VI University Hospital Center, Oujda, MAR; 3 Department of Radiology, Mohammed VI University Hospital Center, Oujda, MAR

**Keywords:** anticoagulation therapy, burkitt lymphoma, cerebral venous thrombosis, hypercoagulability in malignancy, multifactorial thrombosis in cancer

## Abstract

Cerebral venous thrombosis (CVT) is an uncommon but serious complication in pediatric patients with cancer. We report the case of a nine-year-old boy diagnosed with Burkitt lymphoma involving the nasopharyngeal region, who developed superior sagittal sinus thrombosis during chemotherapy. Neurological examination prior to treatment revealed no signs of intracranial hypertension. However, two weeks after completing the first course of chemotherapy, the patient presented with severe headaches and intermittent vomiting. Neuroimaging confirmed cerebral venous thrombosis, while the tumor mass was regressing. Anticoagulant therapy with low-molecular-weight heparin was initiated and carefully adjusted in response to chemotherapy-induced thrombocytopenia. Clinical evolution was favorable, with complete neurological recovery and radiological resolution of the thrombus. This case emphasizes the importance of early recognition of CVT in pediatric oncology, especially when neurological symptoms occur during chemotherapy, even in the absence of prior intracranial hypertension. It also highlights the challenges of anticoagulant management in the context of treatment-related thrombocytopenia.

## Introduction

Cerebral venous thrombosis (CVT) is a rare event in the general pediatric population and remains uncommon even among children with cancer [1,2]. However, oncologic patients present a higher predisposition to thrombotic complications due to cancer-related hypercoagulability, the use of central venous catheters, infections, and the prothrombotic effects of certain chemotherapy agents such as L-asparaginase and corticosteroids.

Burkitt lymphoma, a highly aggressive B-cell non-Hodgkin lymphoma, is treated with intensive multi-agent chemotherapy protocols that further enhance the risk of thrombosis [3]. 

The diagnosis of CVT in children is challenging due to its variable and nonspecific neurological presentation, ranging from headaches and seizures to focal deficits or altered consciousness. Neuroimaging, particularly magnetic resonance venography (MRV), is essential for early diagnosis [4].

Management of CVT in pediatric oncology patients is complex because of the balance between anticoagulation therapy and the risk of bleeding, especially in the context of thrombocytopenia or mucosal damage caused by chemotherapy [5].

Herein, we report a case of cerebral venous thrombosis in a child undergoing treatment for Burkitt lymphoma, and we discuss the current challenges in diagnosis, management, and prevention of thrombotic events in pediatric cancer patients.

## Case presentation

**Figure 1:**
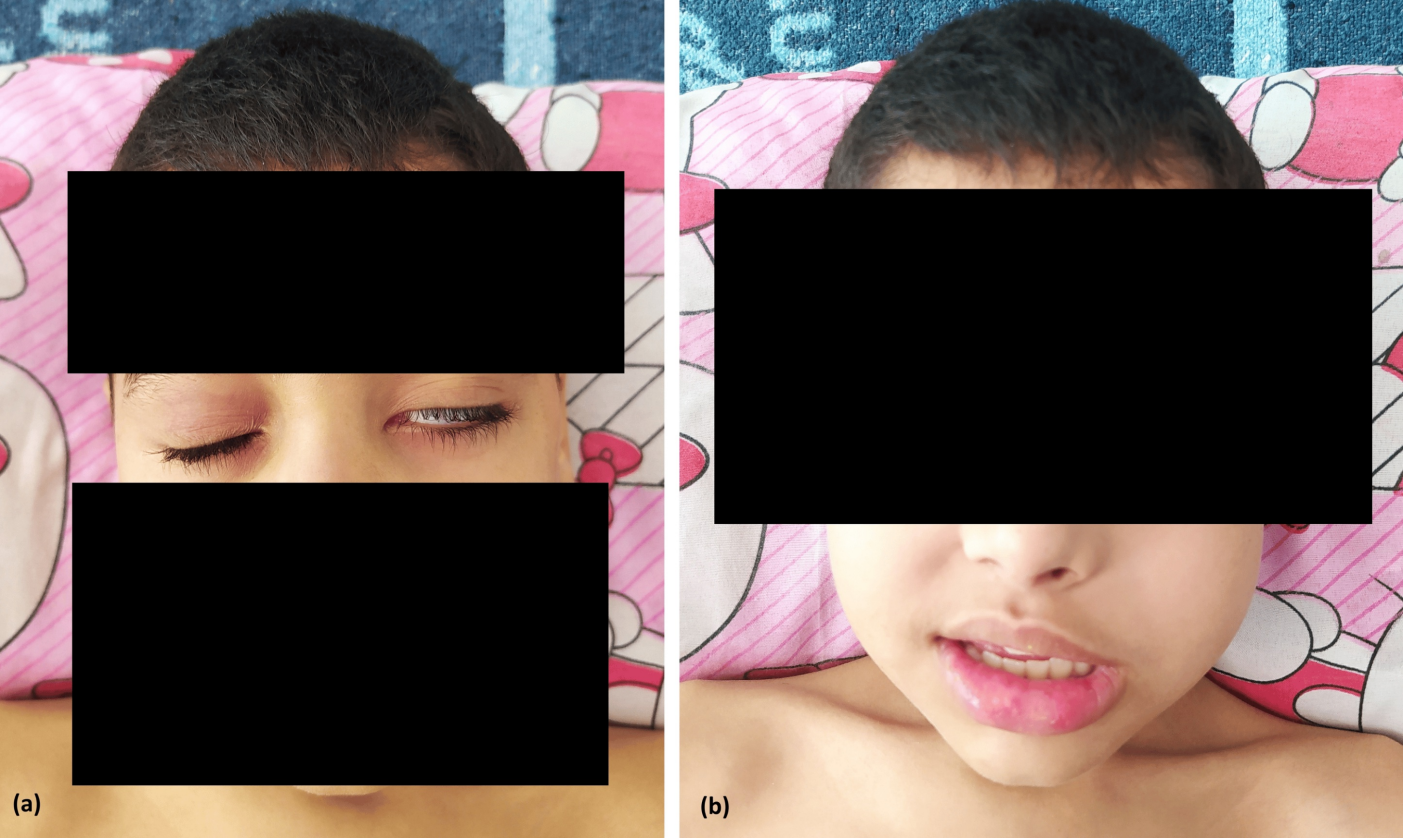
Frontal view showing left peripheral facial palsy. There is an early left lagophthalmos (a), as well as flattening of the left nasolabial fold and drooping of the left labial commissure (b).

We report the case of a nine-year-old boy, blood type A+, weighing 23 kg (−3 standard deviations) for a height of 132 cm (within normal range), with no significant medical history or family history of thrombosis, admitted to our department for the management of Burkitt lymphoma. At initial examination, the child presented with a large nasopharyngeal mass extending to the left submandibular and laterocervical regions, associated with left peripheral facial paralysis and paresis of the ipsilateral upper limb (Figure 1).

**Figure 2:**
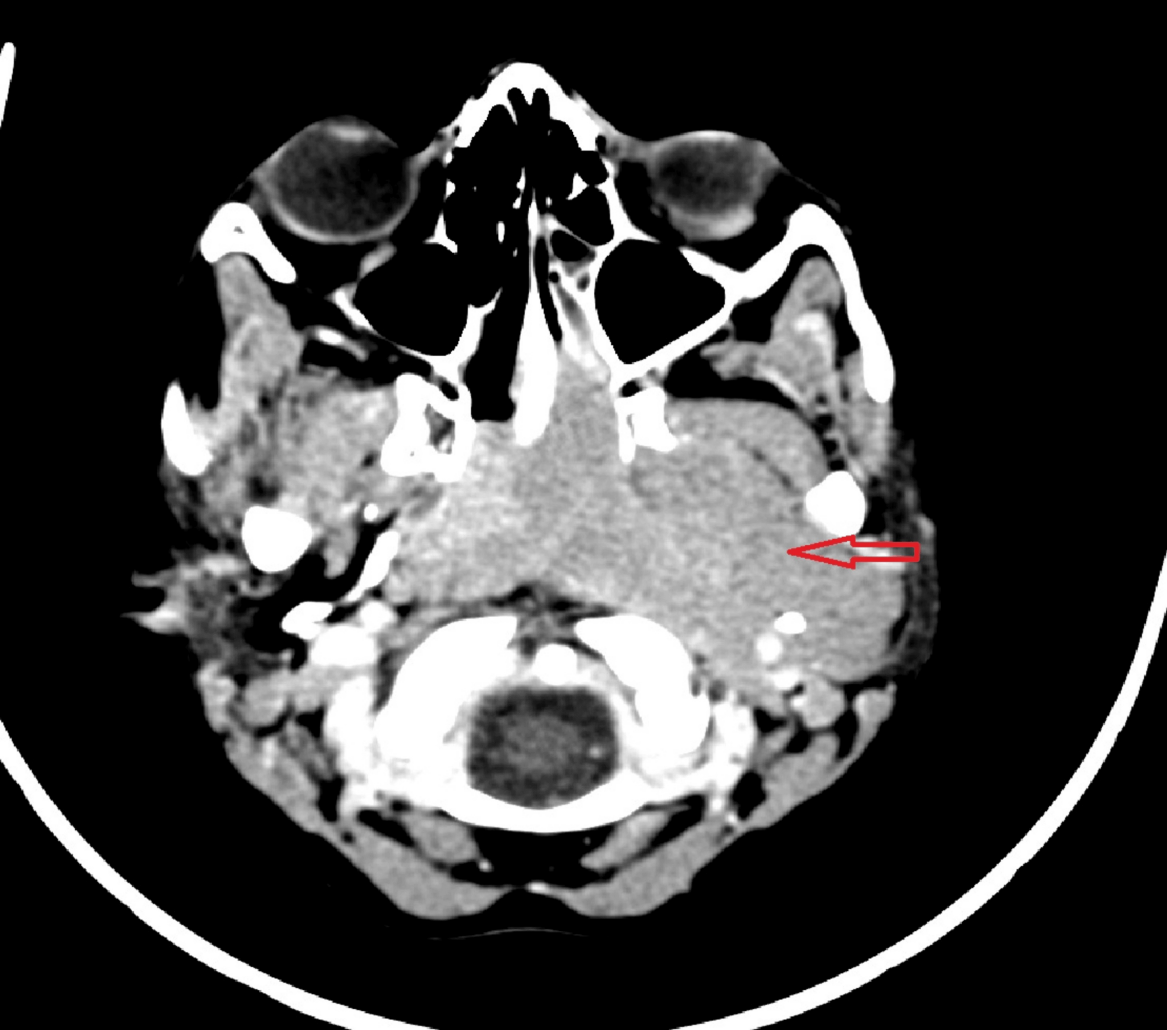
Axial CT image at the level of the nasopharynx showing a large tumor mass (red arrow) extending anteriorly toward the choanae and laterally invading the left masticator space.

Cervico-cerebral computed tomography (CT) and magnetic resonance imaging (MRI) revealed a poorly defined tumoral process centered on the left posterolateral wall of the nasopharynx, with intracranial extension but no involvement of the dural venous sinuses, measuring approximately 70×41 mm, along with ipsilateral laterocervical lymphadenopathy (Figure 2).

**Figure 3:**
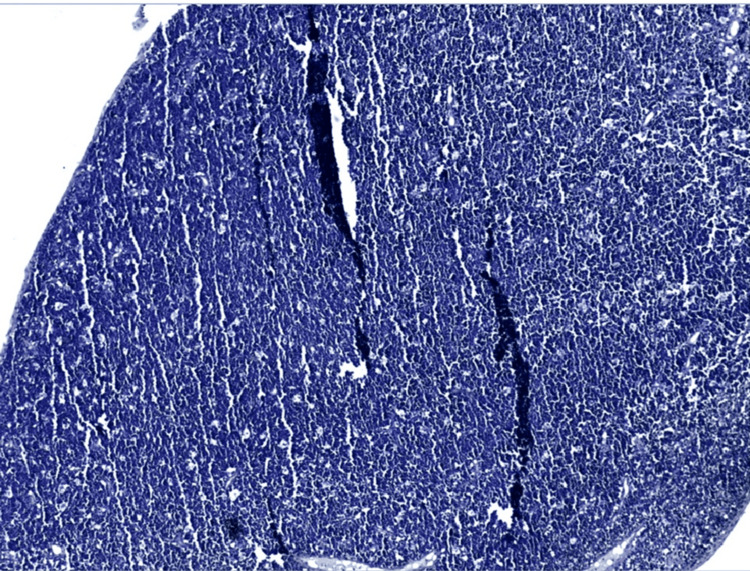
Low power view showing a lymphomatous tumoral proliferation arranged in diffuse sheets with a starry sky appearance (Hematoxylin-Eosin coloration, Magnification x4)

**Figure 4:**
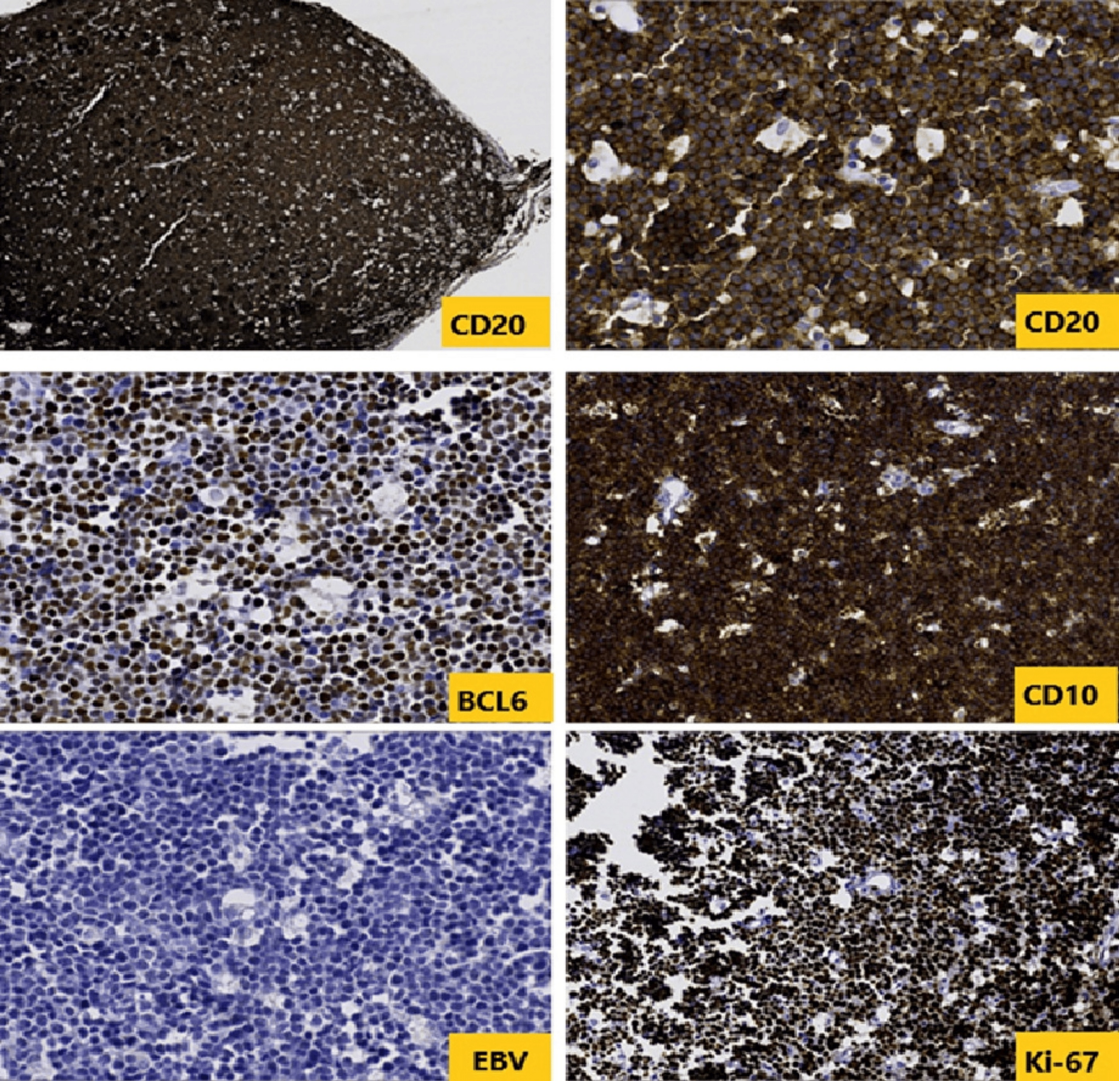
Immunohistochemistry demonstrated diffuse positivity for CD20, BCL6 and CD10, whereas Epstein–Barr virus (EBV) was negative. Ki-67 was estimated at 100%

Histological and immunohistochemical analysis of a nasopharyngeal biopsy confirmed the diagnosis of Burkitt lymphoma (Figures 3, 4).

The initial complete blood count revealed a mild decrease in hemoglobin levels, leukocytosis with neutrophil predominance, and a normal platelet count. Biochemical analysis showed normal urea and creatinine values, a fibrinogen level within the reference range, an elevated erythrocyte sedimentation rate, and a marked increase in lactate dehydrogenase (LDH). The main laboratory findings are summarized in Table 1.

**Table 1:** Summary of the patient’s laboratory findings.

Test	Result	Reference Range (Units)
Hemoglobin	11.4	12.0 – 16.0 g/dL
Leukocytes	13200	4,000 – 10,000 /µL
└ Neutrophils	8870	1,500 – 7,500 /µL
└ Lymphocytes	2610	1,000 – 4,000 /µL
Platelets	243000	150,000 – 450,000 /µL
Urea	0.16	0.10 – 0.50 g/L
Creatinine	3.6	5 – 12 mg/L
Fibrinogen	2.4	2.0 – 4.0 g/L
Erythrocyte Sedimentation Rate	43	< 20 mm (1st hr)
Lactate Dehydrogenase (LDH)	2060	140 – 280 IU/L

**Figure 5:**
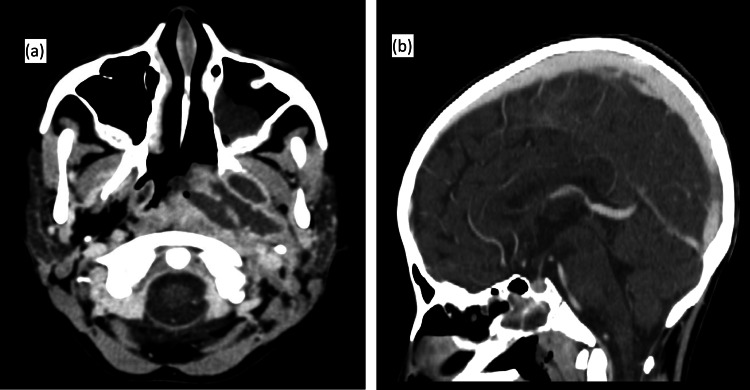
CT images of the patient after the first cycle of chemotherapy (a) Axial CT image at the level of the nasopharynx showing regression in size of the tumoral process along the posterior wall of the nasopharynx. (b) Sagittal CT image with contrast injection demonstrating good patency of the superior sagittal sinus.

Therapeutic management was initiated six days after admission, according to the GFAOP/LMB 2005 protocol, combining intravenous cyclophosphamide with intrathecal injections of methotrexate, cytarabine, and hydrocortisone. A first CT scan of the brain, neck, chest, abdomen, and pelvis showed partial regression of the tumor mass, now largely necrotic, measuring approximately 45×38 mm versus 70×41 mm, with regression of the left paravertebral mass and reduction in size of the pancreatic lesion (Figure 5).

The protocol was continued with intravenous vincristine, cyclophosphamide, and methotrexate, and oral prednisone, along with further intrathecal injections.

**Figure 6:**
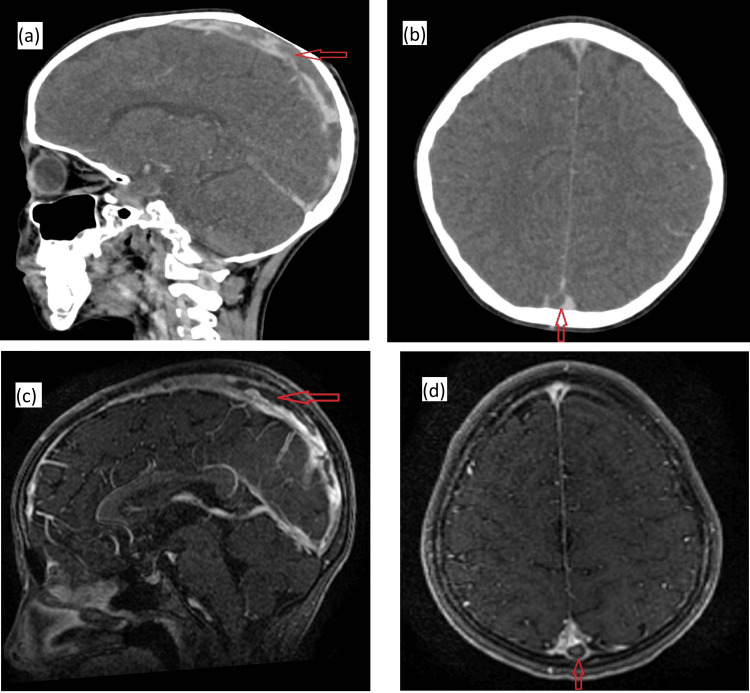
(a) Sagittal CT image with contrast injection showing a hypodense area within the superior sagittal sinus (red arrow), consistent with cerebral thrombophlebitis. (b) Axial CT image with contrast injection showing a filling defect within the superior sagittal sinus corresponding to the empty delta sign (red arrow). (c) Sagittal MRI (Fast Spoiled Gradient Echo (FSPGR) sequence) demonstrating a hypointense signal within the superior sagittal sinus, compatible with thrombosis. (d) Axial MRI (FSPGR sequence) highlighting the empty delta sign (red arrow).

Fourteen days after completion of the first chemotherapy cycle, the child developed severe headaches and intermittent vomiting. Cerebral CT, followed by MRI, revealed a thrombus in the superior sagittal sinus consistent with cerebral venous thrombosis, while the nasopharyngeal mass continued to regress (Figure 6).

No signs of intracranial hypertension had been observed prior to the onset of symptoms, and the patient’s thrombophilia work-up was unremarkable.

Anticoagulation therapy with low-molecular-weight heparin (100 IU/kg every 12 hours) was initiated, with close biological monitoring, particularly of platelet counts, as anti-Xa activity could not be measured due to its unavailability in our hospital. Anticoagulation was maintained without interruption of the chemotherapy protocol. Before each intrathecal injection, two doses of heparin were withheld, along with an additional post-procedure dose, totaling three missed doses over 36 hours. A single prolonged suspension of four days was required during post-chemotherapy aplasia, when the platelet count dropped to a nadir of 5,000/µL. During this period, the patient experienced a single episode of moderate epistaxis, managed effectively with nasal packing and platelet transfusions. Anticoagulation was resumed once the platelet count exceeded 50,000/µL.

**Figure 7:**
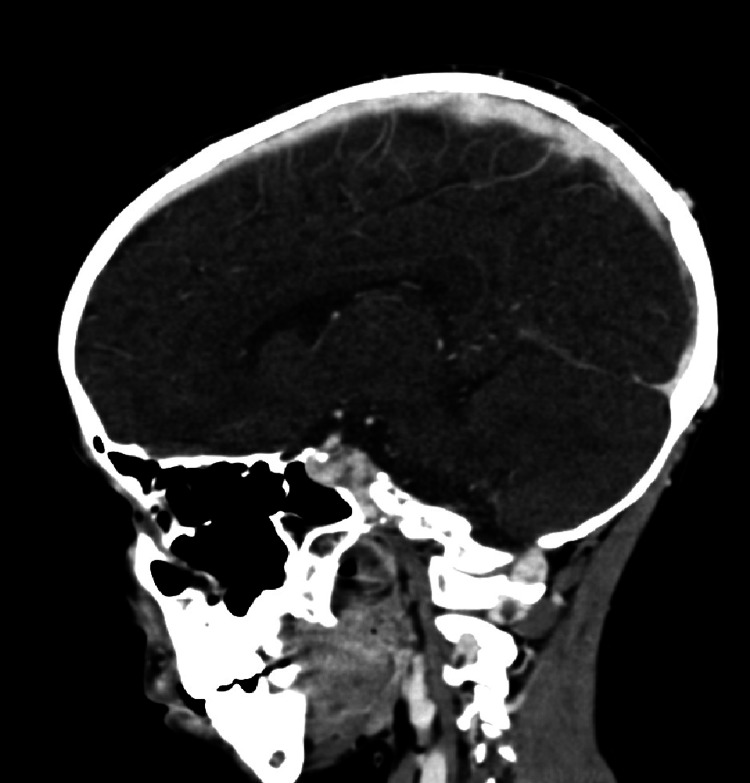
Sagittal CT image with contrast injection, performed two months after the initiation of anticoagulant therapy, showing absence of hypodensity within the superior sagittal sinus, indicative of complete resolution of the thrombosis.

Notably, despite several episodes of chemotherapy-induced aplasia, this patient experienced only one episode of severe thrombocytopenia, which contrasts with the typical hematological profile observed in Burkitt lymphoma patients. Neurological outcome was favorable, with full recovery of left upper limb strength and gradual improvement of facial paralysis following physical rehabilitation. A follow-up CT scan performed two months later confirmed complete recanalization of the superior sagittal sinus (Figure 7).

Anticoagulation therapy was continued for six months, resulting in full resolution without residual thrombus. At the end of chemotherapy, complete remission was confirmed by positron emission tomography (PET scan). At six months follow-up, the child remains free of tumor recurrence or thrombotic events, with no detectable neurological sequelae.

Written informed consent was obtained from the patient’s parents for the publication of this case report and the accompanying images.

## Discussion

Cancer-associated thromboembolic events are increasingly recognized in children, representing a significant complication during oncologic management. Children with malignant diseases have up to a 30-fold higher thrombotic risk compared to the general pediatric population. The majority of these events occur in the venous system, particularly in association with the placement of central venous catheters (CVCs) [2]. Among these manifestations, CVT remains rare in the pediatric cancer population [1]. It is primarily reported in patients with acute lymphoblastic leukemia (ALL) undergoing chemotherapy, with an estimated incidence of approximately 6% [6]. This complication is largely attributed to a hypercoagulable state induced by antithrombin III deficiency secondary to the use of L-asparaginase [7]. In children treated for advanced non-Hodgkin lymphoma, cerebral thrombosis cases have also been reported, although less frequently, with an incidence below 3%, occurring mainly during induction or consolidation phases [7].

Cancer is recognized as a major contributor to hemostatic dysregulation, inducing a multifactorial prothrombotic state. This hypercoagulability is primarily driven by the overexpression of tissue factor (TF) by malignant cells, which initiates the coagulation cascade through complex formation with factor VII. Additionally, cancer cells can damage the vascular endothelium, thereby promoting platelet activation, the release of procoagulant factors, and inhibition of the fibrinolytic system [2]. In children with cancer, this prothrombotic state is further exacerbated by a chronic inflammatory environment. The release of pro-inflammatory cytokines, such as tumor necrosis factor (TNF) and interleukin-1, amplifies TF expression while enhancing platelet aggregation and leukocyte adhesion, ultimately contributing to thrombus formation [8]. In the specific context of cancer-associated CVT, multiple mechanisms are implicated. Beyond hypercoagulability and systemic inflammation, direct vascular compression or infiltration by the tumor, as well as disruptions in natural anticoagulant pathways - particularly involving protein C and thrombomodulin - play key roles in the pathophysiology of this complication [9]. Beyond these biological mechanisms, thrombotic risk in pediatric oncology results from a complex interplay between patient-related factors and therapeutic interventions. Among patient-related factors, age is critical: infants under two years of age are especially vulnerable due to the small caliber of their blood vessels, which increases the likelihood of complications from CVCs. Conversely, adolescents, particularly those older than ten years, also exhibit heightened susceptibility [10]. The type of malignancy is another major determinant. ALL is strongly associated with an elevated thrombotic risk, largely owing to the use of agents such as asparaginase and corticosteroids, which foster a prothrombotic milieu. Additional patient-related factors, such as tumor location, obesity, or the presence of hereditary or acquired thrombophilias (e.g., protein C or S deficiencies, factor V Leiden mutation), are recognized contributors [8]. Notably, non-O blood groups are linked to a higher thrombotic risk, likely due to elevated von Willebrand factor levels and reduced proteolysis by ADAMTS13 [2].

Regarding treatment-related factors, several agents and therapeutic modalities contribute to thromboembolic events. CVCs, commonly used in pediatric oncology, remain a major risk factor. Asparaginase induces hypofibrinogenemia and suppresses natural anticoagulants, thereby promoting thrombosis. Corticosteroids alter coagulation by increasing prothrombin, factor VIII, and von Willebrand factor levels, while also reducing fibrinolysis, thus reinforcing a prothrombotic state [11,12]. Other chemotherapeutic agents, such as anthracyclines, cisplatin, or all-trans retinoic acid (ATRA), have also been linked to increased thrombotic risk through various mechanisms. For example, in female patients with metastatic breast cancer treated with combination chemotherapy (cyclophosphamide, methotrexate, 5-fluorouracil, vincristine, and prednisone), a thrombosis incidence of 17.6% has been reported during treatment, compared with approximately 2% in untreated patients, the majority being venous thromboembolic events [13]. In our case, the identified risk factors included the advanced stage and substantial tumor burden, as well as the non-O blood group (A+ in our patient). Furthermore, the patient received a chemotherapeutic regimen comprising cyclophosphamide, methotrexate, vincristine, and prednisone - closely resembling the protocol previously described, with the exception of 5-fluorouracil. This therapeutic similarity reinforces the hypothesis that the cerebral venous thrombosis observed in our patient was most likely a complication of chemotherapy, particularly given the absence of any prior signs of intracranial hypertension and the documented patency of the dural sinuses before treatment initiation. Although an initial intracranial extension of the tumor could be considered a potential etiology, this hypothesis appears less plausible in light of the absence of clinical manifestations following the prephase treatment and the confirmed patency of the venous sinuses on the initial imaging assessment. Finally, additional clinical or therapeutic conditions such as radiotherapy, surgery, prolonged immobilization, or severe infections in the context of immunosuppression can further amplify this risk. In particular, infections induce a systemic pro-inflammatory state that activates the coagulation cascade [8].

Clinically, cerebral venous sinus thrombosis (CVST) can present with highly variable manifestations, making diagnosis particularly challenging in children. The most frequently reported presentations include seizures, altered consciousness, encephalopathy, and focal neurological deficits such as hemiparesis, unilateral sensory disturbances, or cranial nerve palsies. These signs may be accompanied by diffuse neurological symptoms, such as headaches, nausea, or vomiting [4]. In children, especially in older infants, both focal and diffuse manifestations are commonly observed. In our case, the diagnosis of cerebral venous thrombosis was suspected due to the onset of severe headaches associated with vomiting, occurring in the context of intensive chemotherapy. This clinical presentation is suggestive of intracranial hypertension syndrome, a classic feature of intracranial venous thrombosis [1].

Cerebral CT is generally the first-line imaging modality when CVST is suspected [7]. It may reveal spontaneous hyperdensity within a venous sinus, indicating the presence of a thrombus. Following contrast injection, a characteristic filling defect, known as the “empty delta sign,” can be observed, particularly in the posterior portion of the superior sagittal sinus [14]. However, even with contrast enhancement, CT has limited sensitivity and may fail to detect thrombosis in approximately 40% of cases [4]. MRI is currently considered more sensitive than CT [15]. MRI, particularly T1- and T2-weighted sequences combined with MRV, allows for precise visualization of the thrombus and potential parenchymal complications. In the acute phase, the thrombus appears isointense on T1 and hypointense on T2 sequences, which may be mistaken for normal venous flow. In such cases, MRV plays a crucial role in confirming the diagnosis. In the subacute phase, the thrombus becomes hyperintense on T1, facilitating its identification. The reference imaging techniques for diagnosing CVST are contrast-enhanced CT venography and brain MRI combined with MRV. The diagnosis is based on demonstrating the absence of cerebral venous flow, with or without associated signs of infarction [4]. The superficial venous system is more frequently affected than the deep system, with the most common locations being the transverse, superior sagittal, sigmoid, and straight sinuses.

The reference treatment for CVT in children, including in oncologic settings, is based on the administration of low-molecular-weight heparin (LMWH), in the absence of specific validated recommendations for the pediatric cancer population [5]. Management therefore relies on general guidelines for pediatric thromboembolic (TE) events [16]. LMWH is administered twice daily (1 mg/kg every 12 hours for children older than two months; 1.5 mg/kg for younger infants), aiming to maintain anti-Xa activity between 0.5 and 1.0 IU/mL, measured four to six hours after injection [2,8]. This treatment offers several advantages: no food or drug interactions, prolonged half-life allowing once- or twice-daily dosing, and a lower association with osteoporosis compared to unfractionated heparin (UFH) [16]. Although UFH is still occasionally used, it requires continuous infusion and close biological monitoring due to its short half-life and a higher risk of adverse effects, such as bleeding and osteoporosis [17,16].

Warfarin, an oral anticoagulant of the vitamin K antagonist (VKA) class, is rarely employed in pediatric patients due to its narrow therapeutic index, the absence of an appropriate pediatric formulation, and numerous interactions with food and concomitant medications [2]. Moreover, stringent monitoring of the international normalized ratio (INR) is required to maintain a therapeutic range between 2.0 and 3.0, which poses significant challenges in the context of pediatric oncology, particularly among patients with malnutrition or severe systemic illness. In our patient, LMWH was selected given its established safety profile and widespread use in managing thrombosis within the pediatric population, as well as its minimal interaction with the chemotherapeutic agents administered. During the treatment course, regular hematological monitoring, including platelet counts, was undertaken to promptly identify any potential heparin-induced thrombocytopenia. Notably, anti-factor Xa activity assays were not performed due to their unavailability at our institution. Furthermore, to ensure therapeutic continuity outside the hospital setting, the patient’s mother received training in the proper technique for subcutaneous administration of LMWH.

Direct oral anticoagulants (DOACs), including factor Xa inhibitors (rivaroxaban, apixaban, edoxaban) and the thrombin inhibitor (dabigatran), represent an appealing alternative to conventional anticoagulants. Their antithrombin-independent action, wide therapeutic window, and low potential for food and drug interactions make them particularly attractive in pediatrics [18]. The randomized EINSTEIN-Jr trial demonstrated the efficacy and good tolerability of rivaroxaban in children, including in a cancer subgroup. However, these findings should be interpreted with caution. A smaller observational study by Barg et al. reported a notable incidence of thromboembolic and bleeding complications, particularly among children with high tumor burden [2]. Thus, despite their pharmacological advantages, the safety of DOACs in the pediatric oncologic setting still requires confirmation through larger, dedicated clinical trials.

In children with cancer, anticoagulation management is particularly challenging due to the high frequency of thrombocytopenia, resulting either from the malignancy itself or the myelotoxic effects of chemotherapy. During the acute phase, platelet transfusions are often necessary to maintain platelet counts above 50 × 10⁹/L, the threshold required to continue curative anticoagulation. During the maintenance phase, LMWH dosing is adapted to platelet counts: full dosing is maintained above 50 × 10⁹/L, reduced by half between 20 and 50 × 10⁹/L, and suspended below 20 × 10⁹/L. These therapeutic adjustments aim to balance thrombosis prevention with bleeding risk management [5].

In the oncologic setting, invasive procedures (lumbar puncture, bone marrow aspiration or biopsy, surgery, central venous catheter placement) are frequent and require heightened vigilance. To minimize the risk of bleeding or spinal hematoma, it is recommended to withhold at least two LMWH doses before any elective procedure, particularly lumbar puncture or epidural interventions, and to monitor anti-Xa activity whenever possible prior to the intervention [1,5].

Finally, fondaparinux, another anticoagulant from the LMWH family, is administered once daily (0.1 mg/kg for patients < 50 kg) and may serve as an alternative in selected situations [8]. However, LMWH remains the anticoagulant of choice in pediatric cancer patients due to its proven efficacy, favorable safety profile, and ease of dose adjustment in high-risk clinical situations.

To date, no robust evidence supports the establishment of general recommendations for thromboprophylaxis in children with malignant tumors [8]. Although the increased risk of venous thromboembolic events (VTE) and their potential complications may justify primary prevention, the lack of consensus and solid evidence limits its implementation.

Regarding CVST, although its specific mortality remains below 10%, it frequently results in neurological sequelae-motor, cognitive, or sensory-reported in 17% to 79% of patients at hospital discharge or during follow-up [4]. Grace et al. notably reported that 15% of children with asparaginase-associated CVST developed chronic headaches, while other studies have described visual disturbances, sixth cranial nerve palsies due to intracranial hypertension, or deficits related to venous infarctions [5].

The follow-up of children with CVST must be rigorous, with regular neurological and ophthalmological monitoring to enable early detection of signs of intracranial hypertension. An ophthalmological assessment is recommended during the first year, even in the absence of symptoms. In cases of persistent headaches, nausea, or vomiting, imaging studies are required to rule out complications. Follow-up with MRI or CT venography is typically performed at three, six, and 12 months to evaluate the evolution of the thrombosis (extension, recanalization, or stenosis) [4].

## Conclusions

CVST is a rare but serious complication in pediatric oncology, often linked to cancer-related hypercoagulability and intensive treatments. Early diagnosis through advanced imaging and prompt anticoagulation, mainly with low-molecular-weight heparin, are key to improving outcomes. However, the risk of bleeding, thrombocytopenia, and long-term neurological sequelae remains significant. The absence of clear recommendations for primary thromboprophylaxis underscores the need for further research, while multidisciplinary management is essential to optimize care and prognosis.
